# Null-Cell Ectopic Pituitary Adenoma of the Nasal Cavity

**DOI:** 10.1155/2023/5561092

**Published:** 2023-10-12

**Authors:** Nicholas Figaro, Jibran Juman, Ashton Ramsundar, Fidel Rampersad, Melanie Johncilla, Solaiman Juman

**Affiliations:** ^1^Department of Clinical Surgical Sciences, University of the West Indies, Eric Williams Medical Sciences Complex, Champs Fleur, Trinidad and Tobago; ^2^Department of Radiology, University of the West Indies, Eric Williams Medical Sciences Complex, Champs Fleur, Trinidad and Tobago; ^3^Department of Pathology, Port of Spain General Hospital, Port of Spain, Trinidad and Tobago

## Abstract

An ectopic pituitary adenoma (EPA) is an uncommon type of pituitary adenoma, accounting for only 2% of all pituitary adenomas. EPAs are benign tumors that can occur anywhere along the migratory embryonic path of the pituitary gland and have no relationship to intrasellar elements. They are usually hormonally active and have a minor female predominance. The clinical features of EPAs are highly dependent on its hormonal activity, anatomical location, and its local mass effect. Appropriate radiological imaging is essential for the evaluation of EPAs. Imaging investigations show a normal pituitary gland and sellar turcica, provide details on the size of the tumor, its margins, and extent, and help with surgical planning. The criteria for diagnosing an ectopic pituitary adenoma depend on detailed histopathological examination. EPA management should be individualized. We present a case of a 71-year-old male who presented with a 9-month history of left nasal obstruction, purulent nasal discharge, and intermittent anterior epistaxis. The patient was being managed by his general practitioner for chronic rhinosinusitis but failure of his symptoms to resolve prompted a visit to the otorhinolaryngologist. The patient was diagnosed with a null-cell ectopic pituitary adenoma through histological analysis of a biopsy specimen that showed adenohypophyseal cells without cell-type-specific differentiation. The patient subsequently underwent an endoscopic endonasal excision and had an uneventful hospital stay.

## 1. Introduction

Pituitary adenomas are benign tumors that arise from the pituitary gland and account for approximately 10–15% of all intracranial neoplasms [1]. They are commonly classified based on their size, hormone secreting capacity, and location within the gland. Ectopic pituitary adenomas, in contrast, are uncommon, making up just 2% of all pituitary adenomas [2]. Ectopic pituitary adenomas are completely extrasellar and have no relationship to intrasellar elements [3]. Two thirds of EPA cases are hormonally active; in addition, EPAs can occur anywhere along the migratory embryonic path of the pituitary gland [4]. As a result, the clinical diversity of EPAs can often lead to diagnostic and treatment challenges [5].

Null-cell adenomas (NCAs), first described by Kovacs et al. in 1980, are pituitary adenomas that lack immunohistochemical evidence of distinct cell lineage differentiation for pituitary hormones and transcription factors [6]. NCAs are clinically nonfunctioning pituitary lesions that typically exhibit more aggressive clinical behavior due to their lack of differentiation; as such, NCAs typically present with symptoms of mass effect or are discovered incidentally [6]. In this article, we present a rare case of a null-cell ectopic pituitary adenoma found within the nasal cavity of a 71-year-old male which was successfully removed endoscopically.

## 2. Case Presentation

**Figure 1:**
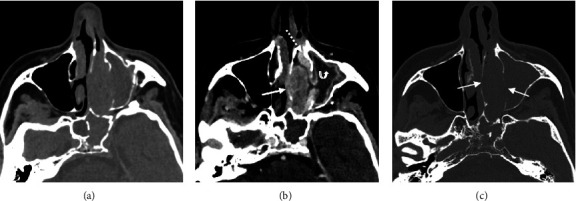
Axial view CT facial bone. (a) Noncontrast image demonstrating soft tissue density opacification of the left nasal cavity and left maxillary sinus with normal aeration of the right nasal cavity and maxillary sinus. (b) Postcontrast image revealing a heterogeneously enhancing mass within the middle left nasal cavity (solid arrow) with homogeneous enhancement of the inferior nasal turbinate (dashed arrow) which is displaced inferiorly and laterally. Mucosal enhancement of the left maxillary sinus with no enhancement of the contents of the left maxillary sinus (curved arrow) indicating retained secretions. (c) Bone reconstruction images showing bowing of the bony nasal septum and medial wall of the left maxillary sinus (solid arrows) which are reduced in density indicating chronicity with no features of bony destruction.

**Figure 2:**
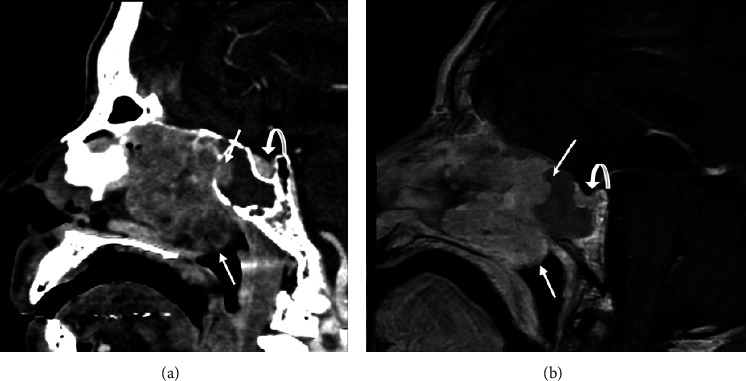
Sagittal views: postcontrast images of (a) CT facial bones and (b) MRI facial bone T1-weighted sequence which reveal the craniocaudal extent of a left nasal cavity heterogeneously enhancing mass with mild protrusion of the posterior border into the sphenoid sinus and superior nasopharynx (solid arrows). The pituitary gland (curved arrows) can also be seen with a similar enhancement pattern to that of the left nasal cavity mass.

**Figure 3:**
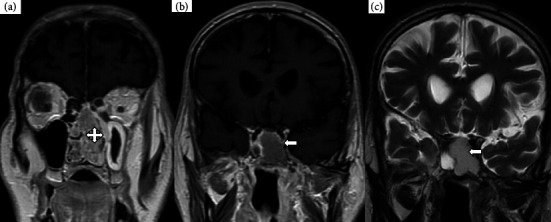
MRI facial bones coronal views: (a, b) postcontrast TI-weighted images and (c) T2-weighted images. The localization of the heterogeneously enhancing mass to the left nasal cavity can be seen (cross arrow). The sphenoid sinus contents (solid arrows) demonstrate a decreased signal intensity relative to the lateral ventricles on T2-weighted images while exhibiting an increased signal intensity on T1-weighted images. These findings align with the presence of retained secretions resulting from the obstruction of the drainage pathway caused by the mass within the left nasal cavity.

A 71-year-old male with a medical history of type 2 diabetes mellitus and hypertension presented with a 9 -month history of left nasal obstruction, purulent nasal discharge, and intermittent anterior epistaxis. The patient was being medically managed by his general practitioner for chronic rhinosinusitis; however, failure of his symptoms to resolve prompted a visit to the otorhinolaryngologist. Examination of the patient with a nasal endoscope revealed a reddish, polypoidal mass occupying approximately two-thirds of the left nasal cavity. Examination of the patient's head, neck, and cranial nerves were unremarkable. The patient's body habitus was normal for his age and he denied any systemic complaints. A contrast-enhanced computed tomography scan and magnetic resonance imaging of the patient's paranasal sinuses revealed a heterogeneously enhancing soft tissue mass extending from the superior nasopharynx to the middle third of the inferior turbinate with no bony erosion (Figures 1–3). The sphenopalatine foramen region, pterygopalatine fossa, and sellar were normal.

**Figure 4:**
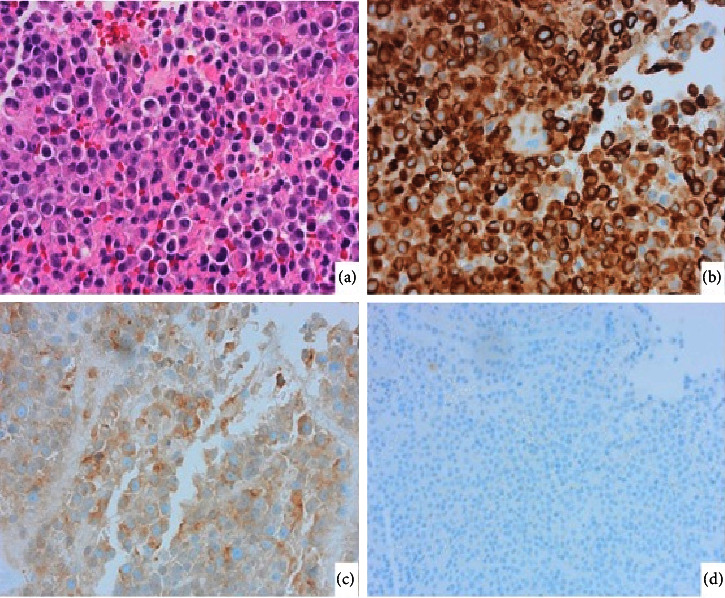
Histology showed sheets of small to medium sized round cells (a) that were positive for keratin (AE1/3) (b) and synaptophysin (c) and negative for calretinin (d).

**Figure 5:**
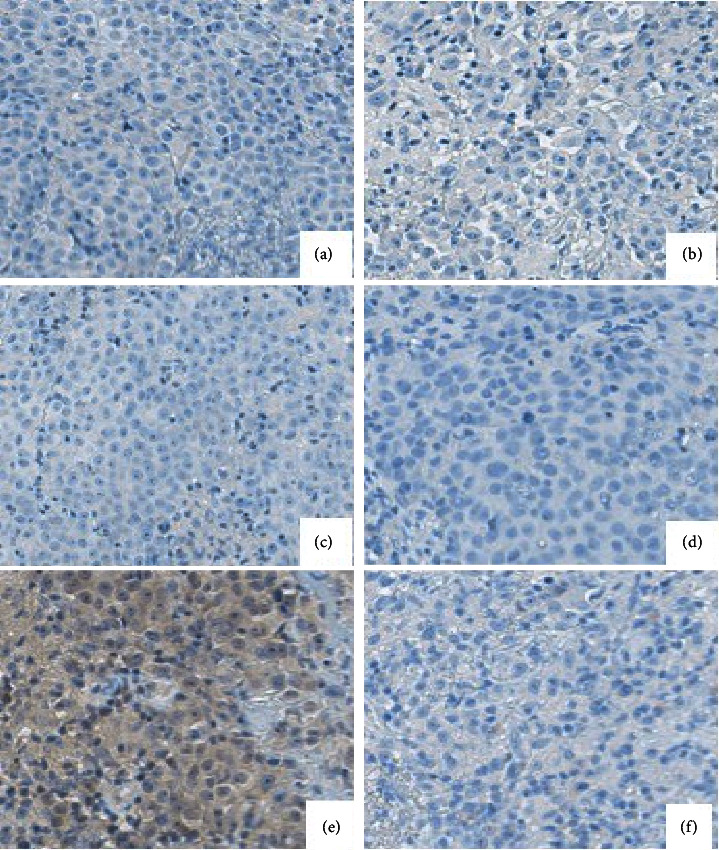
Immunohistochemical stains for subtyping were negative for TSH (a), prolactin (b), LH (c), GH (d), FSH (e), and ACTH (f), respectively.

A biopsy of the nasal mass was performed under local anesthesia. Histological analysis of the biopsy specimen showed sheets of small to medium sized round cells with a mitotic index of 3/10 per high power field, positive for keratin (A1/3) and synaptophysin and negative for calretinin supportive of a pituitary adenoma (Figure 4). The calretinin was specifically performed to rule out an olfactory neuroblastoma as both these entities can be positive for synaptophysin. The negativity of the calretinin further supports that this is an ectopic adenoma as olfactory neuroblastomas are positive for calretinin [7]. A neuroendocrine tumor is yet another tumor that frequently exhibits synaptophysin positivity. Due to the tumor's absence of coarse (“salt and pepper”) chromatin, large nucleoli, and an abundance of eosinophilic to amphophilic cytoplasm, this entity was ruled out. Pituitary adenomas typically have prominent nucleoli and eosinophilic to amphophilic cytoplasm, which are not seen in neuroendocrine tumors [8]. Further immunohistochemical stains for growth hormone (GH), prolactin, adrenocorticotrophic hormone (ACTH), thyroid stimulating hormone (TSH), follicle stimulating hormone (FSH), and luteinizing hormone (LH) were all negative (Figure 5). Endocrinological blood investigations for GH, prolactin, ACTH, TSH, FSH, and LH were all within normal limits. The patient was diagnosed with a null-cell ectopic pituitary adenoma and subsequently underwent an endoscopic endonasal excision under general anesthesia. He had an uneventful hospital stay and was discharged postoperative day one. At the out-patient follow-up, one year after surgery, the patient was asymptomatic and nasal endoscopy showed no residual tumor.

## 3. Discussion

The World Health Organization defines an ectopic pituitary adenoma as a benign pituitary gland tumor located outside the sellar turcica that is not in continuity with the intrasellar pituitary gland [9]. Ectopic pituitary adenomas are believed to originate from residual cells situated along the migratory path of the pharyngeal pituitary, which journeys from Rathke's pouch to the sellar turcica. The initial development of the anterior segment of the pituitary occurs as a pharyngeal outgrowth of epithelial ectoderm, referred to as Rathke's pouch. During approximately the eighth week of gestation, a portion of this structure detaches and migrates via the craniopharyngeal canal into the sellar, ultimately forming the anterior section of the pituitary gland, the site from which the majority of pituitary adenomas originate. It is worth noting that ectopically deposited cells have the infrequent potential to give rise to adenomas at various locations along this migratory pathway [1, 9, 10]. Since its first description by Erdheim in 1909, several ectopic sites have been reported, namely, sphenoid sinus, clivus, parapharyngeal space, nasal cavity, nasopharynx, third ventricle, and suprasellar region [2, 4, 11]. These rare neoplasms have a minor female predominance (1.3 : 1) with most patients presenting in the 6^th^ decade [9]. Approximately, two-thirds of EPAs are located in the sphenoid sinus and suprasellar region [11]. The clinical features of an EPA are highly dependent on its hormonal activity, anatomical location, and its local mass effect [9, 12]. The majority of patients will report symptoms that can be broadly divided into the following three categories: hormonal, neurological, and physiological. Hormonal symptoms are usually depicted as the typical pathologic response to hormones released by tumors. For example, ACTH-secreting adenomas can give rise to Cushing's syndrome, characterized by clinical features such as moon facies, truncal obesity, abdominal striae, and hirsutism. This syndrome is primarily marked by hypercortisolism as indicated by an abnormal dexamethasone suppression test. In contrast, GH-secreting adenomas in adults can manifest as acromegaly, which is characterized by extremity enlargement, coarse facial features such as frontal bossing and prognathism, and associated symptoms such as carpal tunnel syndrome, diabetes, or cardiomyopathy. Laboratory results typically reveal elevated levels of insulin-like growth factor and a failed oral glucose tolerance test. Among adenoma subtypes, prolactinomas account for the majority of cases and may present with symptoms such as gynecomastia, erectile dysfunction, decreased libido, galactorrhea, amenorrhea in females, and elevated prolactin levels [10, 13].

Patients with EPAs positioned at the sphenoid sinus, clivus, and/or suprasellar region frequently experience neurological symptoms such as headaches, visual disturbances (diplopia, loss of visual acuity, and blurring), cerebrospinal fluid leak, and other focal neurological deficiencies (facial paresthesia). In contrast, EPAs found in the nasal cavity or the nasopharynx often cause physiological symptoms such as nasal congestion and epistaxis similar to that of the index case [10, 13]. In our situation, the patient's nonspecific, persistent rhinosinusitis symptoms and the hormonal inactivity of the tumor contributed to the delay in diagnosis.

It is essential that the evaluation of these ectopic tumors be conducted using appropriate radiological imaging. Imaging investigations will not only show a normal pituitary gland and sellar turcica but will also show the size of the tumor, provide details on its margins, and extent and help with surgical planning [9, 11]. Traditionally, MRI shows mild to moderate heterogenous contrast enhancement, without a unique T1- or T2-weighted image intensity, whereas nonenhanced CT demonstrates tumors that are iso to hypodense to grey matter, showing modest enhancement in postcontrast studies [1, 9]. Our case served as a demonstration of both of these findings.

In addition to specific radiological findings, the criteria for diagnosing an ectopic pituitary adenoma depends on detailed histopathological examinations inclusive of specific immunohistochemical markers and the demonstration of pituitary hormone and transcription factors and less on ultrastructural studies [14]. However, null-cell pituitary adenomas or hormone-immune negative adenomas histologically demonstrate adenohypophyseal cells without cell-type-specific differentiation proven via immunohistochemical, pituitary hormone, and transcription factor staining [14]. Null-cell pituitary adenomas are extremely rare, accounting for less than 5% of nonfunctioning pituitary adenomas [15, 16]. Our case proved challenging for the histopathologist as the differential diagnosis for a unilateral nasal mass is extensive with several pathologies having similar clinical presentations. Furthermore, EPAs must be differentiated from other neuroendocrine tumors, such as carcinoids, neuroendocrine carcinomas, site-specific malignant epithelial neoplasm, and metastatic neoplasms [9, 17].

Due to its rarity, there is a lack of experience in EPA treatment [3]. As such, a number of parameters, including clinical symptoms, tumor size, location, degree of invasion, and hormone-secreting type, should be taken into consideration when treating them [3]. Surgical excision is generally considered the cornerstone of treatment, with radiotherapy reserved for cases of incomplete resection [1, 9]. Several novel case studies have also cited success with pharmacological treatment in hormonally active EPA cases [9, 12]. Dopamine agonists such as cabergoline and bromocriptine are now being used as first-line therapy for prolactinomas, while somatostatin analogs such as octreotide and lanreotide, as well as growth hormone (GH) antagonists like pegvisomant, are recommended as the initial treatment for GH-secreting adenomas [10]. These therapeutic agents hold a particular value in cases of more aggressive tumors, including those that have invaded the cavernous sinus, where complete resection poses a considerable risk of bleeding and associated morbidity. In addition, pharmacotherapy can potentially serve as an alternative to surgery, offering the possibility of delaying or even obviating the need for surgical intervention, particularly in elderly patients or individuals burdened by significant medical comorbidities and relative contraindications to surgical procedures [10, 12].

## 4. Conclusion

In conclusion, EPAs are rare neoplasms that can pose significant diagnostic and therapeutic challenges. Due to their location outside of the sellar turcica and often atypical presentations, these tumors are frequently misdiagnosed or overlooked, leading to delayed or inadequate treatment. Early recognition and accurate diagnosis of EPAs require a high degree of clinical suspicion, combined with appropriate imaging studies, endocrine testing, and histopathological analysis.

## Data Availability

The data used to support the findings of this study are included within the article.
